# 

**Published:** 1902-05-15

**Authors:** 


					﻿THE
DENTAL REGISTER
EDITED BY
NELVILLE S. HOFF, D. D. S.,
ANN ARBOR, MICH.
Vol. LV1.	HAY 15, 1902.	No. 5.
PRICE, ONE DOLLAR A A RAJS, JN .ABVAWgfe-
PUBLISHED MONTHLY BY
SAMUEL· A. CROCKER & CO.,
THE OHIO DENTAL AND SURGICAL DEPOT.
I?” to »O W. otli St., Cineiniinti, O.
Entered at the Postoffice at Cincinnati, 0., as second-class mail matter.
				

